# Novel High Efficient Coatings for Anti-Microbial Surgical Sutures Using Chlorhexidine in Fatty Acid Slow-Release Carrier Systems

**DOI:** 10.1371/journal.pone.0101426

**Published:** 2014-07-01

**Authors:** Andreas Obermeier, Jochen Schneider, Steffen Wehner, Florian Dominik Matl, Matthias Schieker, Rüdiger von Eisenhart-Rothe, Axel Stemberger, Rainer Burgkart

**Affiliations:** 1 Klinik für Orthopädie und Sportorthopädie, Klinikum rechts der Isar, Technische Universität München, München, Bavaria, Germany; 2 Institut für Mikrobiologie, Immunologie und Hygiene, Klinikum rechts der Isar, Technische Universität München, München, Bavaria, Germany; 3 Experimentelle Chirurgie und Regenerative Medizin, Klinik für Chirurgie, Klinikum der Universität München, München, Bavaria, Germany; University Hospital of the Albert-Ludwigs-University Freiburg, Germany

## Abstract

Sutures can cause challenging surgical site infections, due to capillary effects resulting in bacteria permeating wounds. Anti-microbial sutures may avoid these complications by inhibiting bacterial pathogens. Recently, first triclosan-resistances were reported and therefore alternative substances are becoming clinically relevant. As triclosan alternative chlorhexidine, the “gold standard” in oral antiseptics was used. The aim of the study was to optimize novel slow release chlorhexidine coatings based on fatty acids in surgical sutures, to reach a high anti-microbial efficacy and simultaneously high biocompatibility. Sutures were coated with chlorhexidine laurate and chlorhexidine palmitate solutions leading to 11, 22 or 33 µg/cm drug concentration per length. Drug release profiles were determined in aqueous elutions. Antibacterial efficacy against *Staphylococcus aureus* was assessed in agar diffusion tests. Biocompatibility was evaluated via established cytotoxicity assay (WST-1). A commercially triclosan-containing suture (Vicryl Plus), was used as anti-microbial reference. All coated sutures fulfilled European Pharmacopoeia required tensile strength and proved continuous slow drug release over 96 hours without complete wash out of the coated drug. High anti-microbial efficacy for up to 5 days was observed. Regarding biocompatibility, sutures using 11 µg/cm drug content displayed acceptable cytotoxic levels according to ISO 10993-5. The highest potential for human application were shown by the 11 µg/cm chlorhexidine coated sutures with palmitic acid. These novel coated sutures might be alternatives to already established anti-microbial sutures such as Vicryl Plus in case of triclosan-resistance. Chlorhexidine is already an established oral antiseptic, safety and efficacy should be proven for clinical applications in anti-microbial sutures.

## Introduction

Surgical site infections (SSI) are still challenging complications after operations despite of established systemic antibiotic prophylaxis today [1]. Reported rates for SSI are usually in the range of 2–5% [2], [3], but can even rise up to 25% e.g. for colorectal surgery [4]. In case of infection, further surgical interventions become necessary followed by a prolongation of hospitalization up to 10 days [2]. In conclusion, this means higher treatment costs of about 50,000 US$ per case on average [5] for the healthcare system as well as an elevated risk profile for the individual patient.

The presence of foreign material increases the risk of infections [6], especially surgical sutures can act as a wick for SSI [7]. So pathogens from the natural skin flora can easily enter wounds via capillary action. After having attached to suture surfaces the pathogens proliferate and are then able to form biofilms difficult to treat [8], [9]. One potential solution approach to prevent this process is the use of anti-microbial coated sutures. At present, all commercially available anti-microbial sutures are exclusively coated with triclosan such as Vicryl Plus, Monocryl Plus, and PDS Plus [10].

*In vitro* studies using triclosan for anti-microbial sutures, e.g. Vicryl Plus, have resulted in a highly efficient defense against various bacterial pathogens [11], [12]. *In vivo* studies for sternum surgery [13], abdominal wall closure [14], and cerebrospinal fluid shunting procedures [15] indicate significantly lower infection rates for surgical interventions using triclosan coated anti-microbial sutures [16]. In contrast, for some indications there is an ongoing controversial discussion [10], [17], such as appendicitis, breast cancer and colorectal surgery [4], [18]–[20].

The main disadvantage of the so far predominantly used antimicrobial triclosan is its wide non-medical use in cosmetics, hygiene- and household products [21]. First research groups reported resistances of bacteria against triclosan and warnings of potential pathogen selections [22], [23]. Therefore, we were looking for new alternative substances and chose chlorhexidine to equip surgical sutures anti-microbially. This antiseptic is effective against a broad spectrum of relevant pathogens including clinically problematic bacteria like *Staphylococcus aureus* [24]. Besides the known spectrum chlorhexidine is already approved in a variety of medical applications as coating for medical devices [25]–[27], skin antiseptic [28], [29] or as well as oral antiseptic [30].

In a first feasibility study we could show that chlorhexidine coated sutures demonstrated high efficacy against *S. aureus* with the disadvantage of drug concentration related cytotoxicity [31]. Anti-microbial sutures must fulfill a balancing act between inhibiting bacteria and sustaining biocompatibility to the surrounding eukaryotic tissue cells.

The aim of this study was therefore the optimization of new chlorhexidine fatty acid coating formulations for anti-microbial sutures in regard to their drug concentration. We achieved high anti-microbial efficacy over several days combined with ISO 10993-5 required biocompatibility by variation of drug concentrations at 11, 22 or 33 µg per cm suture. Anti-microbially coated sutures were evaluated by measuring tensile strength, inhibition zones, and drug release profiles. These investigations were compared to commercially available plain sutures and triclosan containing Vicryl Plus, as anti-microbial reference.

## Materials and Methods

### Surgical sutures

Plain braided sutures (Gunze Ltd., Japan) made of polyglycolic acid (PGA) without the usual fatty acid coating to avoid sewing effects were used for the preparation of the anti-microbial sutures. As reference one triclosan-containing absorbable suture Vicryl Plus (Ethicon GmbH, Germany) and two standard absorbable sutures PGA Resorba (Resorba GmbH, Germany) and Vicryl (Ethicon GmbH, Germany) were used. All sutures used in experiments corresponded to United States Pharmacopeia standard 1 (USP 1).

### Anti-microbial coating solutions

Anti-microbial coating solutions were prepared by dissolving a fatty acid (palmitic or lauric acid) and chlorhexidine in 99.8% ethanol (C. Roth GmbH, Germany (CR)). In order to prepare the solutions for suture coatings having a mass content of 5% (w/w), 395.0 mg of the corresponding fatty acid and the antiseptic drug were dissolved in 10.0 ml (7.9 g) ethanol. In particular, two groups of coating types were produced. Group I (**CL**): Chlorhexidine diacetate (Sigma-Aldrich GmbH, Germany) in lauric acid as drug carrier (CR). Group II (**CP**): Chlorhexidine diacetate in the drug carrier palmitic acid (CR). For each type of coating three solutions with 20%, 40% or 60% (w/w) drug concentration were formulated. The percentage of drug was related to the 5% total mass of substances (drug plus drug carrier) dissolved in coating solutions. Components were prepared by weighing, using the precision balance Atilon ATL-224 (Acculab Inc., Massachusetts, USA).

### Preparation of anti-microbial sutures

Sutures were cut into 40 cm pieces followed, by coating them in a dip process in flasks under aseptic conditions. The closed flasks were placed on a thermo-shaker (Heidolph Instruments GmbH, Germany) for 2 minutes at 35°C and 150 rpm, in order to achieve reproducible coating weights. After drying for at least 2 hours the coating weight was determined by using the precision balance related to the weight of uncoated sutures. The amount of drug normalized per length of suture (µg/cm) was calculated for each concentration (Table 1).

**Table 1:** Prepared anti-microbial sutures and normalized weights of chlorhexidine.

ratio of chlorhexidine in fatty acid (%)	weight of chlorhexidine (mg)	weight of lauric-/palmitic acid (mg)	normalized drug weight (µg/cm)	coating type and abbreviation for coated sutures	
**20**	0.44	1.76	**11**	chlorhexidine-lauric acid	**CL11**
				chlorhexidine-palmitic acid	**CP11**
**40**	0.88	1.32	**22**	chlorhexidine-lauric acid	**CL22**
				chlorhexidine-palmitic acid	**CP22**
**60**	1.32	0.88	**33**	chlorhexidine-lauric acid	**CL33**
				chlorhexidine-palmitic acid	**CP33**

Amount of chlorhexidine on anti-microbially coated sutures after preparation. The mean coating weight of 40 cm suture samples were determined at 2.2±0.2 mg. The same amounts of chlorhexidine were calculated for the coating types (CL, CP) inside coating solutions (n = 10). The weights on coated sutures for drug contents, fatty acid carriers and normalized mean drug weight per cm suture are given above.

### Tensile strength test

For tensile strength testing the uncoated plain PGA sutures (Gunze), PGA Resorba, Vicryl, Vicryl Plus, and exemplarily novel coated sutures CL22, CP22 were measured. The mechanical strength of individual surgical sutures (n = 5) was determined according to the European Pharmacopoeia (Ph. Eur. 7.0/0667: required minimum for USP 1 sutures is 50.8 N) using the tensile testing instrument Zwicki 8253 (Zwick GmbH, Germany).

### Drug release from fatty acid drug delivery coatings

Drug release kinetics of coated sutures were measured over a time period of 96 hours in phosphate-buffered saline (PBS). For this purpose sutures of 2 cm length (n = 3) were eluted in 1 ml PBS inside a thermomixer MHR 23 (HLC-Biotech, Germany) at 37°C and 200 rpm. After 1 h, 3 h, 5 h, 7 h, 24 h, 48 h, 72 h und 96 h, elution media was taken out and replaced by fresh PBS. Amount of released chlorhexidine was measured by absorption at 280 nm in a microplate spectrophotometer Multiskan Go (Thermo Fisher Scientific GmbH, Germany). Measured drug concentrations were normalized to length of suture samples and drug elution profiles were recorded by cumulating the released drug amounts over time. The percentage of released chlorhexidine was calculated referring to loaded drug per cm length.

### Anti-bacterial efficacy of coated sutures via zones of inhibition

Anti-microbial efficacy was determined in Agar plate diffusion tests by using the strain *Staphylococcus aureus* (ATCC 49230), the main pathogen of implant-associated infections [32], [33]. According to CLSI criteria, bacterial suspensions were prepared to 0.5 McFarland standard [34]. On each Agar plate 1 ml of this suspension was uniformly distributed. Coated sutures with 3 cm length (n = 3) were placed onto these inoculated Agar plates and incubated at 37°C for 24 hours. Inhibition zones were measured (in mm), with a calliper perpendicular to the middle of the threads. Novel coated sutures and Vicryl Plus were put on Agar plates every 24 hours with fresh bacterial lawns and incubated overnight. Over the following days we measured the inhibition zones. Analogous to Ming et al. [12] this procedure was repeated by using the same samples for several days to recognize the remaining anti-bacterial activity until no detectable inhibition zone was left.

### Cytotoxicity study

According to ISO 10993-5:2009, mouse fibroblasts L-929 (DSMZ, Germany) were used for in vitro cytotoxicity testing of coated sutures. The evaluation was performed by measuring the metabolic activity of cells in the presence of eluates from coated sutures via a WST-1 cell proliferation assay (Roche Diagnostics GmbH, Germany). Cells were cultivated in the corresponding media (D-MEM 4.5 g/l D-glucose, Biochrom AG, Germany) containing 10% fetal bovine serum at 37°C and 5.0% CO2 in a humidified atmosphere. Pre-cultures started at 10.000 cells/well inside 96 well microtiter plates, incubated for 24 hours in 200 µl D-MEM. Simultaneously, eluates were generated by eluting coated sutures of 1 cm length (n = 7) in 1.5 ml D-MEM for 24 hours on a thermomixer at 37°C and 300 rpm. After 24 hours cell media was swapped with eluates. Finally, after 48 hours referred to the WST-1 protocol the metabolic activity was measured by quantitative detection of formazan at 405 nm in a spectral photometer. Metabolic activities obtained were referred to activity of pure L-929 culture. A metabolic activity of 70% represents the limit to claim “biocompatibility” for medical devices.

### Statistics

Statistical methods were performed by using the student's t-test (Microsoft Excel 2013) with significant level p<0.05. Mean values and standard deviations were calculated from at least 3 measurements. Calculation of mean values from several measurements was accompanied by the Gaussian error propagation law.

## Results

### Reproducibility of anti-microbial sutures

Coating weight of prepared anti-microbial sutures was an important parameter to estimate weights of drugs per unit length. The weight difference of 40 cm sutures in length between uncoated and coated sutures resulted in a mean coating weight at 2.2 mg±0.2 mg (n = 10) independent from drug concentrations used. The triclosan content on Vicryl Plus sutures was 2.7 µg/cm [35], [36].

### Tensile strength test

The quasi-static tensile strength values of all sutures tested - uncoated, commercially and novel coated sutures - proved mean maximum tensile strength values higher than the Ph. Eur. required minimum of 50.8 N for USP1 absorbable surgical sutures (Table 2). The strength values showed a moderate but significant increase between Gunze and PGA Resorba (p<0.05), similar to novel coated sutures CL22 and CP22 exemplarily. Vice versa for Vicryl Plus sutures, a moderate reduction of tensile strength (p<0.05) was observed compared to Vicryl sutures.

**Table 2:** Mean tensile strength values of surgical sutures (USP 1).

Tensile strength	Gunze	PGA Resorba	CL22	CP22	Vicryl	Vicryl Plus
mean F_max_ (N)	73.7	81.6	80.3	75.9	78.3	72.2
± standard deviation (N)	3.0	1.6	1.5	2.2	1.0	2.2

Mean values of the maximum force F_max_ (n = 5) for uncoated, commercial and novel coated sutures CL22 and CP22 in example are given above. The required minimum for USP 1 surgical sutures at 50.8 N according to European Pharmacopoeia, was reached for all tested sutures.

### Drug release from fatty acid drug delivery coatings

**Figure 1:**
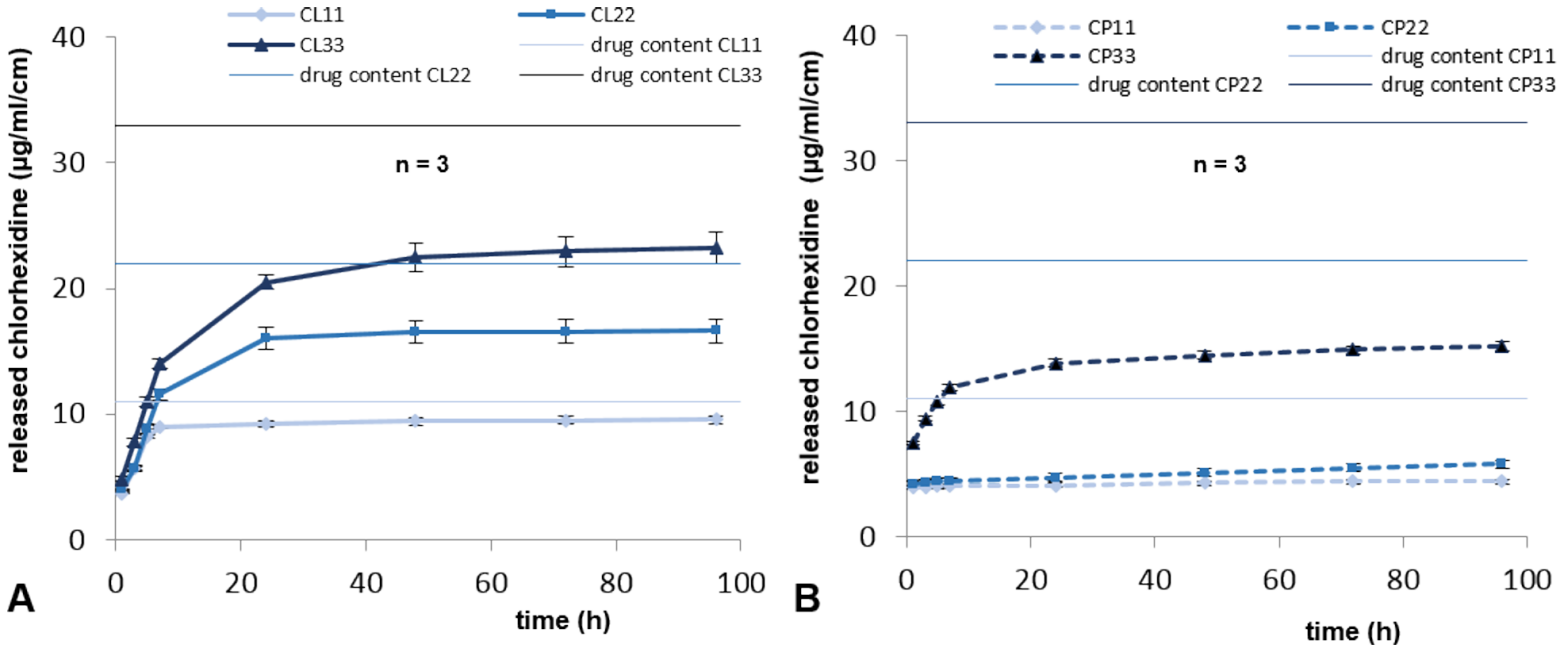
Elution profiles of chlorhexidine coated sutures. Released chlorhexidine in PBS buffer at 37°C for A) chlorhexidine-laurate coatings and B) chlorhexidine-palmitate coated sutures. Elution profiles were determined for each carrier at drug content 11, 22 or 33 µg/cm. The horizontal lines depict the limit of drug release for each concentration, the normalized content of chlorhexidine on coated sutures.

**Figure 2:**
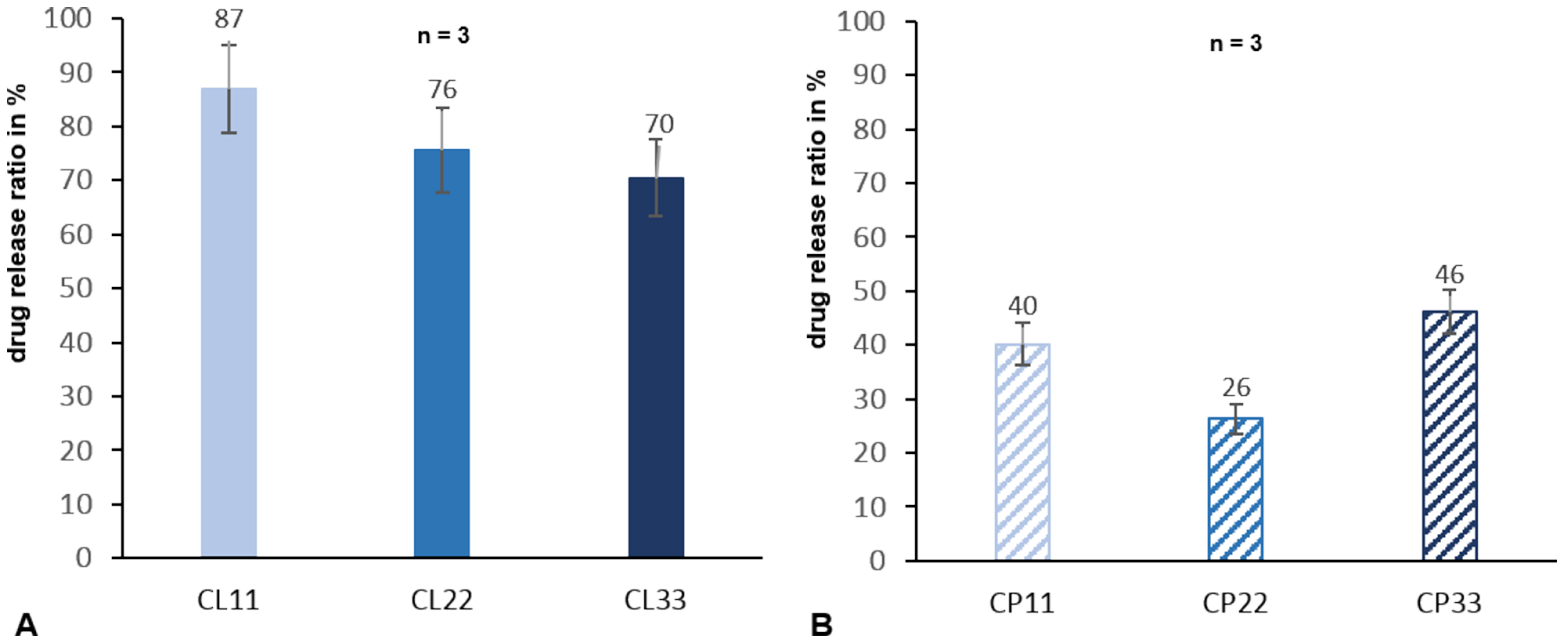
Percentage of chlorhexidine released after 96 Percentage of chlorhexidine release related to the drug content on coated sutures per cm length at 96) chlorhexidine-laurate and B) chlorhexidine-palmitate coated sutures.

Released drug concentrations in PBS eluates normalized by length were determined. All coated sutures demonstrated continuous drug release within four days of experiment. Chlorhexidine was continuously released from lauric acid (Figure 1A) resulting in a drug concentration at 96 hours of 9.6 µg/ml (CL11), 16.6 µg/ml (CL22) and 23.2 µg/ml (CL33). The coating type chlorhexidine in palmitate achieved a drug concentration of 4.4 µg/ml (CP11), 5.8 µg/ml (CP22) and 15.2 µg/ml (CP33) after 96 h (Figure 1B). Percentage of drug release, the ratios between released drugs after 96 hours and amount of drugs on sutures per cm length were compared to each other for chlorhexidine laurate coatings (Figure 2A) and chlorhexidine palmitate coatings (Figure 2B).

### Anti-bacterial efficacy of coated sutures via zones of inhibition

**Figure 3:**
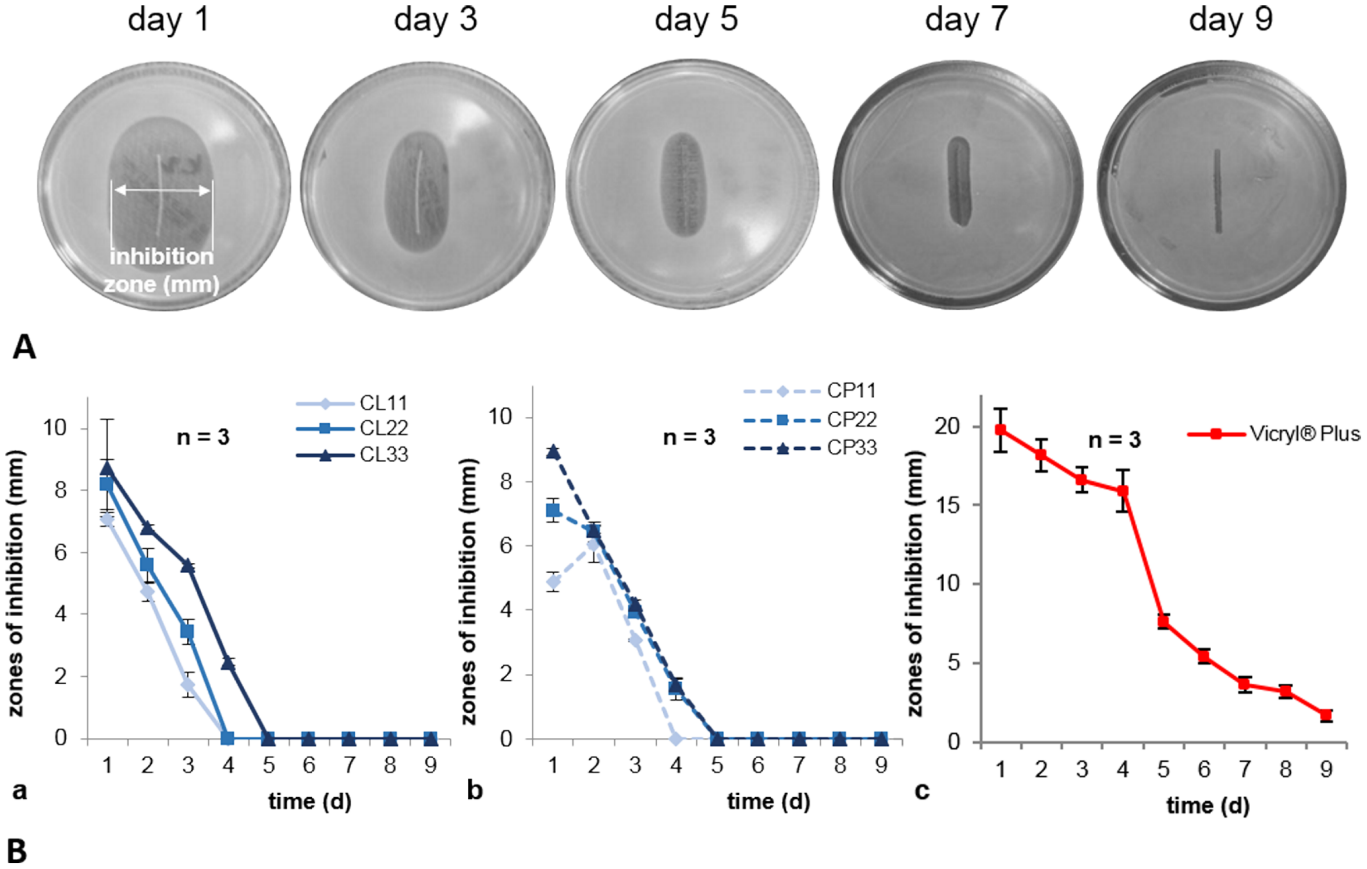
Measuring anti-bacterial efficacy of coated sutures via zones of inhibition. A) Principle of measuring inhibition zones in a longitudinal analysis on the example of a Vicryl Plus suture, in order to achieve anti-microbial effectiveness over several days. B) Anti-microbial efficacies on Agar plates with *S. aureus* lawns (2×10^8^ cfu/ml) for a) chlorhexidine lauric acid and b) chlorhexidine palmitic acid coated sutures. Coated suture samples with three different chlorhexidine concentrations 11, 22 and 33 µg/cm. c) Vicryl Plus as reference for commercial anti-microbial sutures.

Anti-microbial efficacy of coated sutures was daily assessed by using an Agar diffusion assay over several days (Figure 3A). Sutures coated with chlorhexidine in lauric acid (Figure 3B, a) revealed large *S. aureus* inhibition zones after 24 hours of 7.1 mm (CL11), 8.2 mm (CL22) and 8.7 mm (CL33). Inhibition zones during the first three to four days of experiments averaged up to 1.7 mm for CL11, 3.4 mm for CL22 and 2.5 mm for CL33. After the fifth day no inhibition zones were detectable. Chlorhexidine in palmitic acid coatings showed similar results (Figure 3B, b). Inhibition zones after 24 hours were assessed at 4.9 mm (CP11), 7.1 mm (CP22) and 8.9 mm (CP33). The dimensions and durations of the zones of inhibition were dependent on drug concentrations. CP11 ended up after fourth day of experiment, whereas CP22 and CP33 ended up after the fifth day of experiment. The triclosan containing Vicryl Plus showed large inhibition zones after 24 hours on Agar plates with 19.8 mm (Figure 3B, c) lasting for at least nine days and ending up with 1.7 mm zones. The bioavailability of the antiseptics from the fatty acid delivery systems was corroborated by the microbial experiments.

### Cytotoxicity study

**Figure 4:**
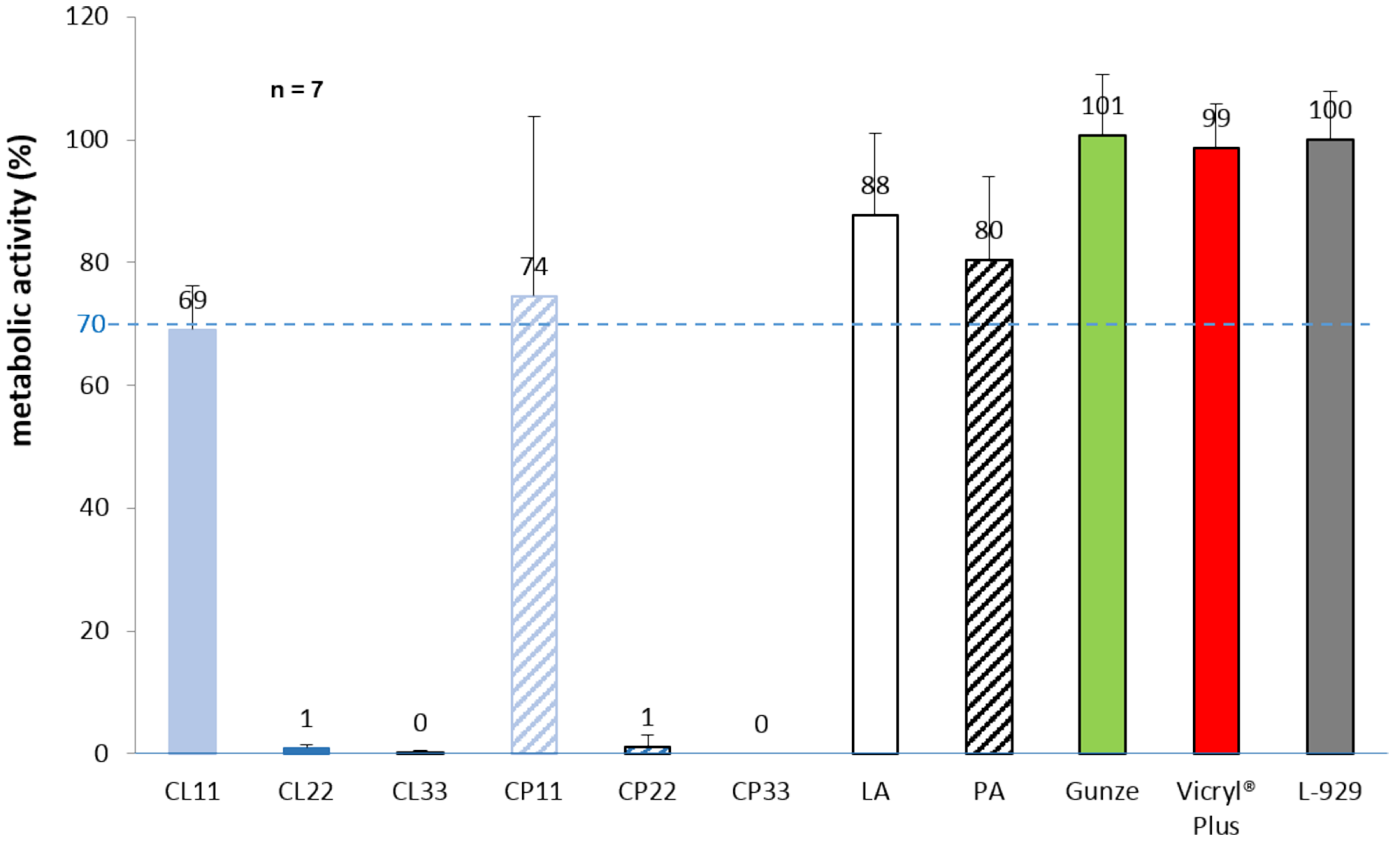
Evaluation of biocompatibility of coated sutures in cytotoxicity tests via WST-1 assay. Metabolic cell activity of fibroblasts in the presence of eluates from coated sutures measured with the WST-1 proliferation assay. Cells were incubated with eluates from coated sutures, suture references: lauric acid (LA), palmitic acid (PA), uncoated suture (Gunze), and Vicryl Plus. All values referred to cellular growth control, pure L-929 mouse fibroblast cultures. Dashed line at 70% pictures the level for acceptable lowering of metabolic activity according to ISO 10993-5:2009 in order to declare biocompatibility of medical devices.

Cytotoxicity tests were performed via eluates from individual coated sutures and references. All metabolic activities were referred to L-929 cell samples used as growth reference without sutures. Coatings with chlorhexidine in lauric acid ended with metabolic cell activities at 69.1±7.0% (CL11), 0.9±0.5% (CL22) and 0.3±0.3% (CL33). Chlorhexidine in palmitic acid coatings showed activities at 74.5±29.3% (CP11), 1.1±1.9% (CP22) and no more cell activity for CP33 sutures. The fatty acid coated references identified metabolic activities at 87.8±13.2% and 80.4±13.4% (lauric acid, palmitic acid). Eluates from uncoated sutures reached a metabolic activity at 100.8±9.7% (Gunze). Vicryl Plus eluates demonstrated activities at 98.7±7.1% (Figure 4).

## Discussion

Surgical site infection still poses a major complication in surgery. Sutures can cause so called suture-associated infections, induced by proliferation of adhering pathogens. Adhering bacteria enter wounds by capillary action and form infamous biofilms, leading to chronic infections [8]. Anti-microbial coatings for surgical sutures can solve that problem via protecting sutures by inhibiting bacterial growth.

In the present study we developed new anti-microbial suture coatings based on fatty acid carriers using chlorhexidine and adjusting their drug concentrations. The aim was to identify anti-microbial sutures posing effective protection against microbes while being biocompatible in regard to eukaryotic cells. Fatty acids constitute a lubricating film and are state of the art in order to reduce the unwanted sewing effect of sutures. This kind of drug-release system still allows slow-release properties, because of low solubility of fatty acid carriers in aqueous environments [37], [38].

In a reproducible dip coating process we developed several coating types at various concentrations based on chlorhexidine with lauric or palmitic acid (CL, CP). Coated sutures obtained were tested systematically regarding their tensile strength, drug release, anti-microbial efficacy against *S. aureus*, and cytotoxicity using a WST-1 assay.

Tensile strength of coated PGA sutures was just negligibly influenced by the dip coating process using ethanol. All coated sutures undergoing this process showed much higher maximum strength values than required by the Ph. Eur. standards for USP1 resorbable sutures. The mean strength values of novel coated sutures were comparable to commercially available PGA sutures (PGA Resorba, Vicryl, Vicryl Plus). Therefore, no negative influence of coatings on in vivo degradation time for anti-microbial coated PGA sutures is to be expected. Flexibility of coated sutures remained steady, no delamination was observed after mechanical stress tests. Consequently, no delamination is to be expected while pulling them through the tissue.

Released drug concentration in PBS for chlorhexidine coated sutures showed a continuous drug release for 96 hours with initial rapid release slowing down significantly after 7 hours. In general, drug release should be as slow as possible, however, anti-microbially effective to inhibit pathogens as long as possible, to achieve long-term protection of the coated biomaterial. Drug release is strongly dependent on drug concentration inside coatings. Referring to drug carriers, palmitic acid coatings showed slower drug release values over time than lauric acid coatings with similar anti-microbial efficacies. Moreover, lesser amounts of chlorhexidine were released from palmic acid coatings. Therefore, anti-microbial effects of coated sutures using palmitic acid carrier should have a higher potency for long-term protection in vivo than coatings using lauric acid carriers.

Novel coated sutures showed high anti-microbial efficacy in agar diffusion tests against *S. aureus*. Anti-bacterial tests on agar plates mimic the tissue contact transferring substances by diffusion. All coated sutures generated inhibition zones for more than 24 hours and documented efficacies over several days, similar to Vicryl Plus. Inhibition zones of CP11, on the second day, showed a little increase, presumably a consequence of non-uniform contact of suture samples on Agar surfaces and therefore diffusion problems. The release of chlorhexidine indicated by the inhibition zones is faster during the first days compared to triclosan, because of its much higher solubility in aqueous environments like PBS or Agar. This faster consumption of substances on sutures leads to earlier leaching of inhibition zones. On the one hand, this could be a benefit, because a sufficient release of antiseptics in the wound area in the first days might be an important factor to prevent a potential early wound infection. The long-term efficacy of chlorhexidine coated sutures against *S. aureus* lasted up to 5 days at high levels. Regarding drug concentrations, the dimension of inhibition zones over time did not differ greatly. Thus, even the low drug content of 11 µg/cm can almost be as effective as 22 or rather 33 µg/cm in protecting surgical sutures, without depletion of the anti-microbial drug on sutures.

Biocompatibility studies on coated sutures demonstrated acceptable cytotoxicities only for the lowest drug concentrations at 11 µg/cm independent from the fatty acid used. Such sutures fulfilled the at least required 70% remaining metabolic activity of L-929 cells to claim non-cytotoxicity according to ISO 10993-5:2009. Nevertheless, those sutures still have a high anti-microbial efficacy. Therefore, 11 µg/cm chlorhexidine coated sutures are potential candidates for further pre-clinical and human in vivo studies. In general, a strong dose-dependent effect for anti-microbial coated sutures was recognized regarding cytotoxicity. To improve biocompatibility a fine tuning with reduction of drug concentration, i.e. from 11 to 9 µg/cm without sacrificing the high anti-microbial efficacies seems promising.

There are limitations of our study, at first, the use of only one bacterial strain, *S. aureus*, for testing efficacy of coated anti-microbial sutures. In vitro tests should be performed to further prove efficacy against other relevant types of pathogens. Second, no effects on biofilms by anti-microbial coated sutures were investigated but bacterial cultures on agar and in suspensions are most common for first evaluation. For that purpose in vitro experiments with microbiological biofilm models are necessary. Third, other antiseptic substances should be identified and investigated regarding biocompatibility and anti-microbial efficacy.

To sum up, we demonstrated a prototypical coating process to provide anti-microbial sutures at high reproducible quality. Mechanical strength tests indicated negligible influences of the coating process comparable to commercial sutures and beyond the Ph. Eur. required strength values for resorbable sutures. Drug release from the novel coated sutures in aqueous media revealed to be dependent on dose, and the entity of the fatty acid carrier. We identified that all used novel chlorhexidine coated sutures proved high anti-bacterial efficacy. Duration of inhibition zones on Agar plates was dependent on the chlorhexidine dose, however there seemed to be no influence from fatty acid carriers. Biocompatibility testing of coated sutures also indicated strong dose dependency.

## Conclusions

In this study we developed novel chlorhexidine coatings for anti-microbial surgical sutures with three different antiseptic concentrations based on palmitic and lauric acid carriers. We demonstrated their high anti-microbial efficacy against *S. aureus* in vitro. In particular, chlorhexidine coated sutures with 11 µg/cm concentration proved acceptable cytotoxicity according to ISO 10993-5 and simultaneously high anti-microbial protection over several days. Such coated sutures represent an alternative in the case of triclosan-resistance for prophylactic sutures. The aim is to support surgeons with an effective weapon to reduce suture-associated surgical site infections. However, further pre-clinical and clinical trials are necessary to confirm safety and efficacy in vivo.
